# Implications of high level pseudogene transcription in *Mycobacterium leprae*

**DOI:** 10.1186/1471-2164-10-397

**Published:** 2009-08-25

**Authors:** Diana L Williams, Richard A Slayden, Amol Amin, Alejandra N Martinez, Tana L Pittman, Alex Mira, Anirban Mitra, Valakunja Nagaraja, Norman E Morrison, Milton Moraes, Thomas P Gillis

**Affiliations:** 1 HRSA, BPHC, Division of National Hansen's Disease Programs, Laboratory Research Branch, Molecular Biology Research Department @ School of Veterinary Medicine, Louisiana State University, Baton Rouge, LA, USA; 2 Rocky Mountain Regional Center of Excellence, Department of Microbiology, Immunology & Pathology, Colorado State University, Fort Collins, CO, USA; 3 Leprosy Laboratory, Department, Tropical Medicine Institute Oswaldo Cruz-FIOCRUZ, Rio de Janeiro, RJ, Brazil; 4 Center for Advanced Research in Public Health, CSISP, Area de Genomica y Salud, Valencia, Spain; 5 Department of Microbiology and Cell Biology, Indian Institute of Science, Bangalore, India; 6 Center for Tuberculosis Research, Department of Medicine, Division of Infectious Diseases, Johns Hopkins School of Medicine, Baltimore, MD, USA

## Abstract

**Background:**

The *Mycobacterium leprae *genome has less than 50% coding capacity and 1,133 pseudogenes. Preliminary evidence suggests that some pseudogenes are expressed. Therefore, defining pseudogene transcriptional and translational potentials of this genome should increase our understanding of their impact on *M. leprae *physiology.

**Results:**

Gene expression analysis identified transcripts from 49% of all *M. leprae *genes including 57% of all ORFs and 43% of all pseudogenes in the genome. Transcribed pseudogenes were randomly distributed throughout the chromosome. Factors resulting in pseudogene transcription included: 1) co-orientation of transcribed pseudogenes with transcribed ORFs within or exclusive of operon-like structures; 2) the paucity of intrinsic stem-loop transcriptional terminators between transcribed ORFs and downstream pseudogenes; and 3) predicted pseudogene promoters. Mechanisms for translational "silencing" of pseudogene transcripts included the lack of both translational start codons and strong Shine-Dalgarno (SD) sequences. Transcribed pseudogenes also contained multiple "in-frame" stop codons and high Ka/Ks ratios, compared to that of homologs in *M. tuberculosis *and ORFs in *M. leprae*. A pseudogene transcript containing an active promoter, strong SD site, a start codon, but containing two in frame stop codons yielded a protein product when expressed in *E. coli*.

**Conclusion:**

Approximately half of *M. leprae's *transcriptome consists of inactive gene products consuming energy and resources without potential benefit to *M. leprae*. Presently it is unclear what additional detrimental affect(s) this large number of inactive mRNAs has on the functional capability of this organism. Translation of these pseudogenes may play an important role in overall energy consumption and resultant pathophysiological characteristics of *M. leprae*. However, this study also demonstrated that multiple translational "silencing" mechanisms are present, reducing additional energy and resource expenditure required for protein production from the vast majority of these transcripts.

## Background

Bacterial pseudogenes are inactivated, presumably nonfunctional genes that can accumulate in the genomes of bacterial species, especially those undergoing processes such as niche selection or host specialization [1,2]. When a bacterial gene is under low selection pressure, it undergoes a period of frequent nucleotide substitutions because deleterious mutations are not efficiently purged. These mutations can cause the accumulation of in-frame stop codons, reading frame-shifts, or removal of traditional translational start codons or vital sections of the gene, giving rise to a pseudogene [3]. Mutations that destroy promoter or regulatory sequences can result in the "silencing" of transcription or translation or premature termination of protein synthesis [4].

The case of pseudogenes in *Mycobacterium leprae*, an obligate intracellular bacterium and etiologic agent of leprosy, is very dramatic. Its ~3.3 Mb genome consists of 1,614 open reading frames (ORFs) and 1,133 pseudogenes [5]. The pseudogenes represent 41% of the total genes ; the largest percentage found in any bacterial genome sequenced to date .

The overall G+C content of *M. leprae*'s genome is 57.8% [5]. This is 8% lower than that of its close relative *M. tuberculosis *[6], a feature usually described in species undergoing low selection pressure [7,8]. Interestingly, the G+C content of pseudogenes (56.5%) is lower than that of its ORFs (60.1%).

Pseudogenes are distributed throughout the *M. leprae *chromosome and are assigned to the majority of functional groups [5,9,10]. If these genes are indeed nonfunctional in *M. leprae*, they should no longer be required for survival in the specialized intracellular niche in which *M. leprae *resides. Therefore, the study of *M. leprae *pseudogenes is important to expand our understanding of the evolution of this obligate intracellular parasite and to establish the role that pseudogenes play in *M. leprae*'s unique metabolism and parasitism.

Recently, pseudogene transcripts in *M. leprae *have been identified [11,12]. However, the extent of pseudogene transcription and the potential impact that these transcribed genes have on *M. leprae *have not been analyzed critically. The cost of expressing non-functional ORFs could be especially dramatic for *M. leprae *because the speed of pseudogene deletion appears to be slower than in other bacteria [13].

To address this, a study of the overall pseudogene transcriptional profile of *M. leprae *using a global *M. leprae *DNA microarray and reverse transcriptase-PCR analyses was conducted. The results demonstrated that a large number of *M. leprae *pseudogenes were transcribed during growth in the nude mouse foot pad, a model for lepromatous leprosy in man. Analyses of these transcribed pseudogenes using bioinformatics tools and *in vitro *methods demonstrated that potential mechanisms for transcription of these pseudogenes were associated with their residing within gene clusters or downstream of functional ORFs. In addition some of these genes contained functional promoters within their 5' upstream sequence. Since translation of this large number of pseudogenes could have a major impact on *M. leprae's *resources and energy consumption without apparent benefit to its survival and growth, mechanisms for translational "silencing" of these transcripts were also investigated using bioinformatics tools. Results demonstrated that the vast majority of these pseudogenes are "silenced". The "silencing" of these transcripts was found to be associated with: 1) the lack of a strong Shine-Dalgarno (SD), ribosomal binding site, in the 5'-UTR of the majority of these genes; 2) the lack of traditional translational start codons; 3) the presence of multiple in-frame stop codons; and 4) high Ka/Ks ratios indicating low functionality of putative protein products. These data indicated that the majority of pseudogenes were nonfunctional, inactivated genes. However, when one pseudogene containing a functional promoter, SD site, a traditional start codon, a very low Ka/Ks ratio, and encoding 3/4 of its *M. tuberculosis *ortholog was tested for protein production in *E. coli*, the predicted product was observed.

## Results

### Transcriptome of M. leprae

Transcriptional analysis of *M. leprae *ORFs and pseudogenes using the *M. leprae *DNA microarray demonstrated that that 1,353 transcripts had a mean signal to noise ratio (SNR) cutoff value ≥ 2 (raw data are accessible through GEO Series accession # GSE17191 study at: ). Several genes, positive on only 1 of 4 arrays were further analyzed and found positive using RT-PCR analysis (Additional File 1). Therefore the current transcriptome of nude mouse footpad-derived *M. leprae *consists of a total of 1,353 transcripts (Additional File 1). RT-PCR analysis was also used to validate the transcription of 20.5% of gene transcripts positive on the array. The transcriptome represents 49% of the total 2,747 genes surveyed and 867/1,353 (64%) transcripts were from ORFs or protein coding genes from a variety of functional gene categories (Additional File 1). Approximately 11% of these ORFs have previously been shown to produce proteins in armadillo-derived *M. leprae*, however no protein product was observed for any pseudogene in these studies (Additional File 1) [14,15]. The present study demonstrated that 486/1,353 (36%) of the gene transcripts detected were from pseudogenes, demonstrating that 43% of all pseudogenes found in the *M. leprae *genome were transcriptionally active (Additional file 1). This represents the largest number of transcriptionally-active pseudogenes reported to date.

**Figure 1:**
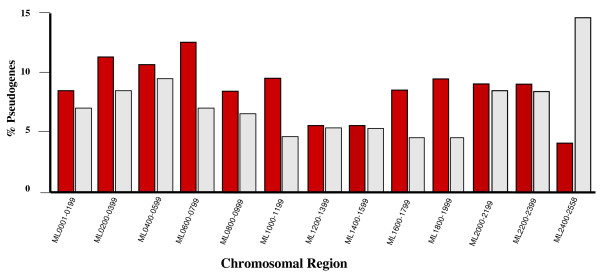
**Distribution of transcribed pseudogenes within specific regions of the *M. leprae *chromosome**. This graph represents the distribution of transcribed pseudogenes as a percent of the total number of pseudogenes within each specific region of the *M. leprae *chromosome (red) and the distribution of pseudogenes within each specific region of the *M. leprae *chromosome as a percent of the total number of pseudogenes within the genome (gray). .

**Figure 2:**
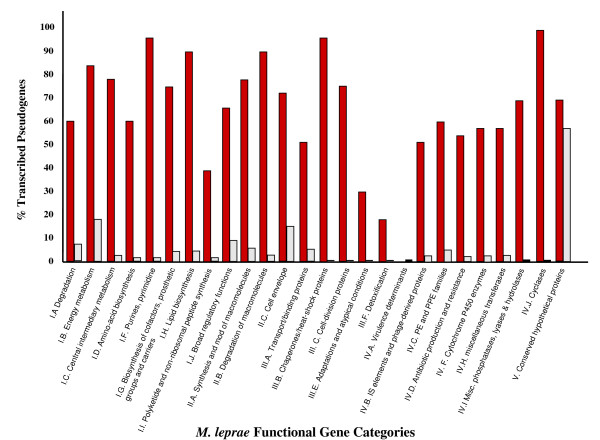
**Distribution of transcribed pseudogenes in functional gene categories of *M. leprae***. This graph represents the distribution of transcribed pseudogenes as a percent of the total number of pseudogenes within each functional gene category of *M. leprae *(red) and the distribution of pseudogenes within each functional gene category as a percent of the total number of pseudogenes within the genome (gray). .

These transcribed pseudogenes were randomly distributed throughout the chromosome (Fig. 1; Additional File 1) and found in 26 of the 29 functional gene categories containing pseudogenes (Fig. 2). The largest number of transcribed pseudogenes 184/486 (38%) was found in the Functional Category V (Conserved Hypotheticals), which contains 776 genes and includes the largest number of pseudogenes in the *M. leprae *genome.

### Read-though transcription of pseudogenes

A plausible explanation for pseudogene transcription in *M. leprae *is read-through transcription as a result of their location within operons or downstream of transcribed ORFs. A total of 112 operons have been identified in the *M. leprae *TN strain (GeneChords-). However, their location within gene clusters has not been fully mapped. Results showed that 10% of transcribed pseudogenes were found within 20 gene clusters (Table 1) and an additional 10% were located downstream of transcribed ORFs (data not shown). These data suggested that 20% of all the pseudogenes could be potentially transcribed by read-through transcription.

**Table 1:** Transcribed *M. leprae *pseudogenes within gene clusters.

**ML#^1 ^Pseudogene**	**Gene Cluster#^2^**	**ML# in Cluster**
ML0212	10	ML0211 – ML0214
ML0477c	26	ML0475 – ML0483
ML0479c	26	ML0475 – ML0483
ML0480c	26	ML0475 – ML0483
ML0495	28	ML0491 – ML0501
ML0496	28	ML0491 – ML0501
ML0499c	28	ML0491 – ML0501
ML0511c	29	ML0510 – ML0523
ML0534	30	ML0532 – ML0537
ML0545c	31	ML0540 – ML0548
ML0546c	31	ML0540 – ML0548
ML0547	31	ML0540 – ML0548
ML0585c	36	ML0582 – ML0587
ML0586	36	ML0582 – ML0587
ML0629	38	ML0624 – ML0633
ML0780	42	ML0778 – ML0782
ML0832	43	ML0831 – ML0833
ML1197	57	ML1195 – ML1200
ML1456c	67	ML1452 – ML1468
ML1457c	67	ML1452 – ML1468
ML1585c	73	ML1581 – ML1598
ML1588c	73	ML1581 – ML1598
ML1595	73	ML1581 – ML1598
ML1662	77	ML1658 – ML1664
ML1693	79	ML1691 – ML1696
ML1771c	83	ML1768 – ML1800
ML1772c	83	ML1768 – ML1800
ML1850c	86	ML1840 – ML1895
ML1851c	86	ML1840 – ML1895
ML1852c	86	ML1840 – ML1895
ML1866c	86	ML1840 – ML1895
ML1868c	86	ML1840 – ML1895
ML1870c	86	ML1840 – ML1895
ML1871c	86	ML1840 – ML1895
ML1874c	86	ML1840 – ML1895
ML1875c	86	ML1840 – ML1895
ML1882c	86	ML1840 – ML1895
ML2212	95	ML2211 – ML2230
ML2214	95	ML2211 – ML2230
ML2216c	95	ML2211 – ML2230
ML2218	95	ML2211 – ML2230
ML2220c	95	ML2211 – ML2230
ML2225c	95	ML2211 – ML2230
ML2328c	98	ML2326 – ML2330
ML2701	108	ML2697 – ML2713
ML2702	108	ML2697 – ML2713
ML2711c	108	ML2697 – ML2713

^1 ^*Mycobacterium leprae *TN, complete genome sequence: .

^2 ^GeneChords, gene cluster analysis: .

**Figure 3:**
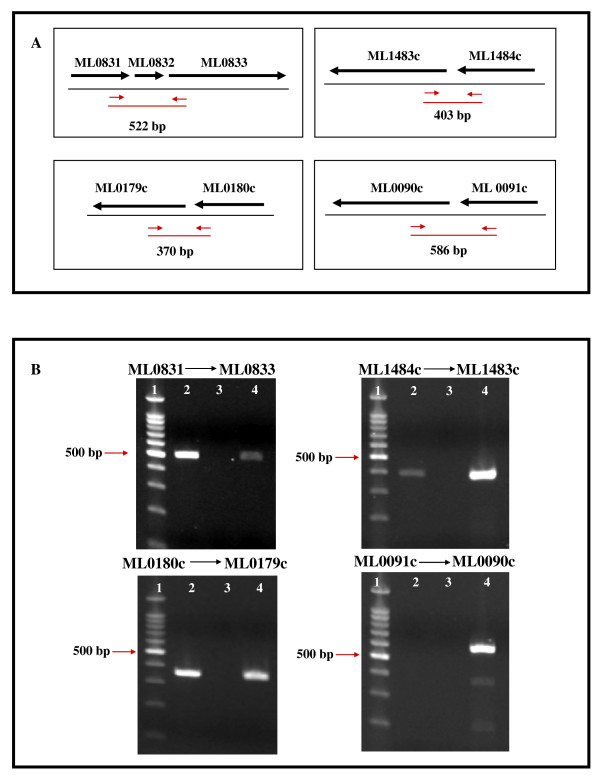
**Read-through transcription of *M. leprae *pseudogenes**. This figure represents the results of RT-PCR analysis of transcriptional read-through between *M. leprae *pseudogenes and their upstream ORFs. Panel A shows mapped genomic regions where pseudogenes (ML0832, ML1483c, ML0179c, and ML0091c) and upstream ORFs are located . Black arrows indicate direction of transcription. Small red arrows indicate forward and reverse primers for RT-PCR. Red line below primers and (bp) designate size of predicted PCR product if genes are expressed as a polycistronic mRNA. Panel B shows ethidium bromide-stained agarose gel analysis of these PCR amplicons: Lane 1, 100 bp DNA ladder (New England Biolabs); Lane 2, PCR amplicons from nu/*nu *footpad-derived *M. leprae *Thai-53 cDNA for each gene set; Lane 3, RT (-) control; Lane 4, 1 ng *M. leprae *DNA (positive control).

When *M. leprae *pseudogenes were experimentally analyzed for their presence within a polycistronic mRNA containing an upstream transcribed ORF, three of four gave the predicted RT-PCR fragment (Fig. 3) and sequencing of the PCR amplicons confirmed their predicted mRNA sequence (data not shown). Fig 3, Panel A depicts the chromosomal location of these pseudogenes, ORFs and primers used for PCR amplification; Panel B depicts results of agarose gel analysis of PCR products generated from *M. leprae *cDNA of these pseudogenes and their respective ORF cDNA. These data demonstrated the presence of a single mRNA transcript containing the predicted RT-PCR products from ML0831–ML0832-(pseudogene)-ML0833, ML1484c-ML1483c (pseudogene) and ML0180c-ML0179c (pseudogene). However, positioning of a pseudogene directly downstream from a transcribed ORF did not guarantee its transcription via a read-through mechanism since no read-through transcript of the predicted length was detected in the cDNA of ML0091c-ML0090c (Fig. 3B) even though individual gene transcripts were detected using microarray analysis (Additional File 1), indicating these genes were transcribed as independent genes.

### Identification of intrinsic stem loop structures

Intrinsic terminators between genes can stop transcript elongation and thus prevent read-through transcription. Hence the 3'UTR and coding regions of upstream transcribed ORFs of transcribed pseudogenes were analyzed for intrinsic stem loop terminator structures. The genomic ΔG_cutoff _for stem loop structures in the *M. leprae *TN genome was previously calculated to be -14.35 [16]. Therefore, only those ORFs which have stem loop structures downstream of the stop codon with ΔG values of < -14 were considered to contain potential intrinsic terminators. Using this criterion, only 27% of ORFs in the *M. leprae *genome contained intrinsic terminators in their 3'UTRs, demonstrating that the majority of *M. leprae *ORFs lack intrinsic terminators (Additional File 2). In addition, only 1.5% of transcribed ORFs upstream of transcriptionally active pseudogenes were found to contain stem loop structures with the potential to act as intrinsic terminators (Table 2). Interestingly, a strong putative intrinsic terminator (ΔG value = -21.84) was found within the 3'UTR of ML0091c, suggesting a potential mechanism for the lack of read-through transcription of the ML0090c pseudogene analyzed above. In contrast a strong intrinsic terminator was found within the coding sequence of ML0180c however, its presence did not stop read-through transcription of the downstream pseudogene ML0179c.

**Table 2:** Prediction of intrinsic stem loop terminators in the 3'-UTR of transcribed ORFs located upstream of transcribed pseudogenes.

**ML#^1^**	***M. leprae *Chromosomal Location**	**Stem Loop ΔG^2^**
ML0090c (p)^3^	112884–111660	
ML0091c	113863–113153	-21.84
		
ML0252	328067–331741	-20.38
ML0253 (p)	331780–332529	
ML0254 (p)	332923–333664	
		
ML0281	363432–364121	-16.11
ML0282 (p)	364131–364909	
		
ML0464	561808–562095	-14.43
ML0465 (p)	562650–563430	
		
ML0533	646543–647835	-20.94
ML0534 (p)	647832–648321	
		
ML0641c (p)	774443–773607	-21.16
ML0642c	775945–774506	
		
ML0644	777176–780127	-17.65
ML0645 (p)	782071–782519	
		
ML0664	800116–800379	-15.2
ML0665 (p)	800705–801966	
		
ML0679	814145–814372	-22.57
ML0680 (p)	814992–816275	
		
ML0727c (p)	870011–869612	-37.21
ML0728c	870918–870028	
		
ML0779	921772–924544	-21.89
ML0780 (p)	924814–925329	
		
ML0928	1098053–1098337	-15.05
ML0929 (p)	1098587–1099186	

^1 ^*Mycobacterium leprae *TN, complete genome: .

^2 ^ΔG < -14 = intrinsic terminator using Genome Scanner for Terminators (GeSTer).

^3 ^(p) = pseudogene.

### Pseudogene promoters

**Figure 4:**
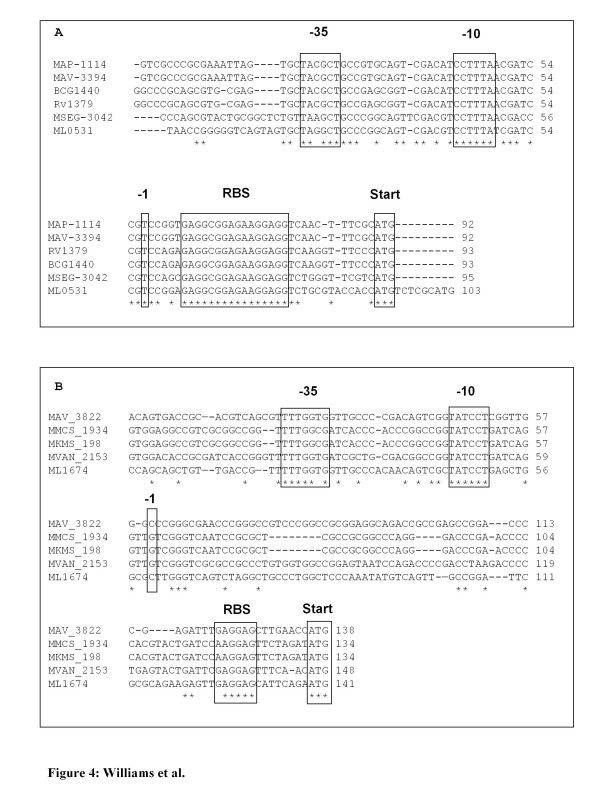
**Promoter-like sequences upstream of transcribed *M. leprae *pseudogenes**. This figure shows representative alignments of promoter-like sequences for *M. leprae *pseudogenes and their mycobacterial homologs. Panel A & B represent the *pyrR *(ML0531) and *rpmB *(ML1674) upstream promoter-like regions containing -35 and -10 regions and initiation site (I) in relationship to their ribosomal binding sites (RBS) and translational start codons (Start), respectively. * indicates identical nucleic acids in all strains. Mycobacterial species abbreviations : MAP-*Mycobacterium avium, subsp paratuberculosis *K-10. MAV-*Mycobacterium avium 104*. BCG-*Mycobacterium bovis *BCG Pasteur 1173P2. RV-*Mycobacterium tuberculosis *H37Rv. MSEG-*Mycobacterium smegmatis *MC^2 ^155. ML-*Mycobacterium leprae *TN. MMCS-*Mycobacterium MCS*. MKMS-*Mycobacterium KMS*. MVAN-*Mycobacterium vanbaalenii *PYR-1

**Figure 5:**
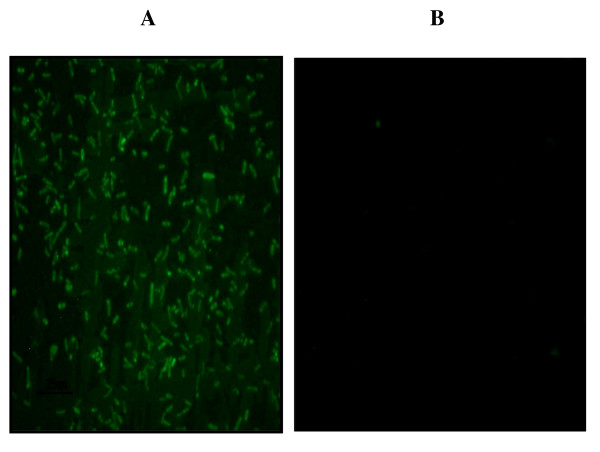
***In vitro *promoter activity in *pyrR *(ML0351) pseudogene**. Panel A shows an image of GFP-fluorescent *E. coli*::*p*Glow-TOPO-TA/ML*pyrR *prom as a result of transformation of *E. coli *XL-1 Blue cells with the *p*Glow-TOPO-TA (promoterless and lacking a SD site) containing the upstream region of the *M. leprae pyrR *pseudogene; including the promoter, SD and start codon (Additional File 6, Fig. 3A) fused into the ATG of *gfp*. A clone containing the ampicillin-resistant phenotype and positive for the *M. leprae pyrR *promoter insert by PCR/DNA sequencing was analyzed by fluorescent microscopy using a Nikon Eclipse E400 fluorescent microscope using a GFP filter (excitation/emission maxima of 480 nm/560 nm). Panel B shows an image of *E. coli*::pGlow-TOPO-TA re-circularized vector (negative control for **background **fluorescence).

The presence of promoter-like sequences in the 5'UTR of transcribed *M. leprae *pseudogenes with translational start codons was investigated using "bend-it" DNA curvature analysis, alignment of promoter-like regions with that of mycobacterial homologs, and *in vitro *confirmation of promoter activity by cloning putative promoters into an *E. coli *promoter-less *gfp *expression-reporter vector. The presence of predicted promoter-like regions with strong upstream DNA static curvature between 9–16.8 deg/turn/peak were observed for 15/92 (16%) of these transcribed pseudogenes (Additional File 3). These promoters also aligned very well with that of other mycobacterial homologs (Table 3). Fig. 4 shows representative promoter-like structures for two of these pseudogenes in relationship to their initiation site, SD sequence, and translational start codon and aligned to that of homologous genes of other mycobacterial species. The function of 10 of these putative promoters was confirmed in *E. coli *promoter-*gfp *fusions by fluorescent microscopy (Table 3). Fig. 5 depicts a GFP positive clone for the promoter region of *pyrR *ML0531.

**Table 3:** Analysis of *M. leprae *pseudogene promoters in silico and in vitro promoter in *E. coli *analyses.

**Pseudogene^1^** **Mycobact** **Ortholog^2^**	**Distance Upstream of Start Codon**	**Intrinsic** **Peak Height^3^**	**Protein Bend** **Peak Height^3^**	**Promoter Sequence (5'3')^4^** **-35 -10 (+1)**	***ML gfp *Express^5^**
ML0086c	-77	10.4	9.5	**AATCAG**ccagagcaggcgagcaaa**CTGAAT--**acagtcccg**T**	+
Rv3817				**TATGCG**ccaggacaagcgagcaag**CCGAAT--**acggtgccg**T**	
					
ML0211	-100	5.8	5.6	**TCACGT**cgaattgcaccgtgtcgg**CCTTAA**atct---agct**A**	+
Rv3627				**TCACGT**cgaattggcacgcgtcgg**CCTTCA**gatcagagtgc**A**	
					
ML0357c	-86	6.5	6.5	**GTTGCT**ggaattc-acactagaac**GTGTTA**--atcagcaag**A**	+
Rv3504				**GTCGCT**ggattcagagactagaac**GTGTTA**caaccgggaag**A**	
					
ML0531	-44	5.5	7.8	**TAGGCT**gcccggcagtcgacgt**CCTTTA**tc-----gatccg**T**	+
Rv1379				**TACGCT**gccgagcggtcgacat**CCTTTA**ac-----gatccg**T**	
					
ML0585c	-31	8	8	**GCCAAC**acgatgtggggatggaAGAGGTct-----ggtcgt**G**	+
Rv1454c				**GCCAAA**acgacgcgcggatgga**AGACGT**cc-----ggccgg**G**	
					
ML0684	-128	6.8	8.6	**ACGCTG**gcgctcatgaccgcgttgc**AGCCTA--**ccgtatcg**C**	+
Rv3339				**ACGCTG**gcgctcatgaccgactcga**AGCCTA--**gcgcatgg**C**	
					
ML1295c	-89	5.6	8	**CGGTGA**cagtcatactgtcaaga**TACCTC**atcccgaaccgg**T**	+
Rv 2138				**CGGTGA**cggtcatgcccagagaa**TACCTC**-tggagtaccat**T**	
					
ML1674	-81	7.8	7.2	**GTGGTT**gcccacaacagtcgc**TATCCT**gag-------ctgg**C**	+
MUL1983				**GTGCTT**gccggggac-gtcgg**TATCCT**agg-------acgg**C**	
					
ML2282c	-92	7.5	6.2	**TGTGTAgctttcgcgacggattTACAGT**cc-----gctcc**cA**	+
Rv0566				**TGCGTA**gctttcgcgacggatt**TACAGT**cc-----gctccc**A**	
					
ML2521	-102	9	9	**ATGACA**aagtggtcgatcacatgc**CCGATC--**accagcaat**T**	+
Rv0310				**ATGCCC**gatcggtcgatcagctgg**CCGATC--**aacaacagc**T**	
					
ML0416	-103	7.8	9	**GTTAAA**aacgtgtttaagagtt**GAAGAG**gg-----ggttaa**C**	ND
MAV4335				**GTTAAG**accttgtttaggagtt**GAAGAT**cg-----gtttaa**C**	
					
ML0459	-89	6.8	11	**ACCTTC**aggtcgccaccgagcg**TGAACG**ct-----ccggat**G**	ND
Rv2616				**ACGGTC**aggtcgccgccgaggg**TCAACG**tt-----ccggat**G**	
					
ML1335c	-56	6.7	10.2	**TACACT**tcggtttctaatctgtg-ga**ATCCAT--**ggcagtc**A**	ND
Rv2090				**TAAACC**tcggcgtcgaatcggcgaga**ACCCAT--**gtcagccA	
					
ML1503c	-105	9.8	9	**AGTGCC**gcgtctacttgctcatc**AGTTAG**cac----agcca**T**	ND
Rv1160				**AGTGCG**gcgtcgacctgctcatc**CGTTAA**cac----agccaT	
					
ML2325c	-110	6.2	7.1	**GTGCAG**tttagggcgatcg**TAAGCG**cggcgct--------t**G**	ND
Rv3711				**GTTGGA**ttcagggcgatcg**CAAGCT**cggcgct--------t**G**	

^1^*Mycobacterium leprae *gene #: .

^2^Mycobacterial orthologous gene: Rv = *M. tuberculosis *H37Rv .; MUL = *M. ulcerans *.; and MAV = *M. avium *.

^3^Bend-it analysis of pseudogenes.

^4^Alignment of mycobacterial promoters performed using ClustalW: .

^5^PCR-amplified promoter-like regions of *M. leprae *genes (Additional File 6) were cloned into *p*Glow-TOPO-TA promoterless reporter-*gfp *vector and expressed in *E. coli *XL1-Blue Supercompetent cells. (+) = positive for GFP expression using fluorescent microscopy.

^6^ND = not done

### Translational start codons in transcribed pseudogenes

Potential mechanisms for translational "silencing" of pseudogenes transcribed in *M. leprae *were analyzed *in silico *using bioinformatics tools. Results demonstrated that 363/486 (75%) of transcribed pseudogenes lacked traditional translational start codons (AUG, GUG, UUG), greatly reducing the translation potential of transcribed pseudogenes into protein products (Additional File 4).

### Ribosome binding strength of the SD regions of transcribed pseudogenes

**Figure 6:**
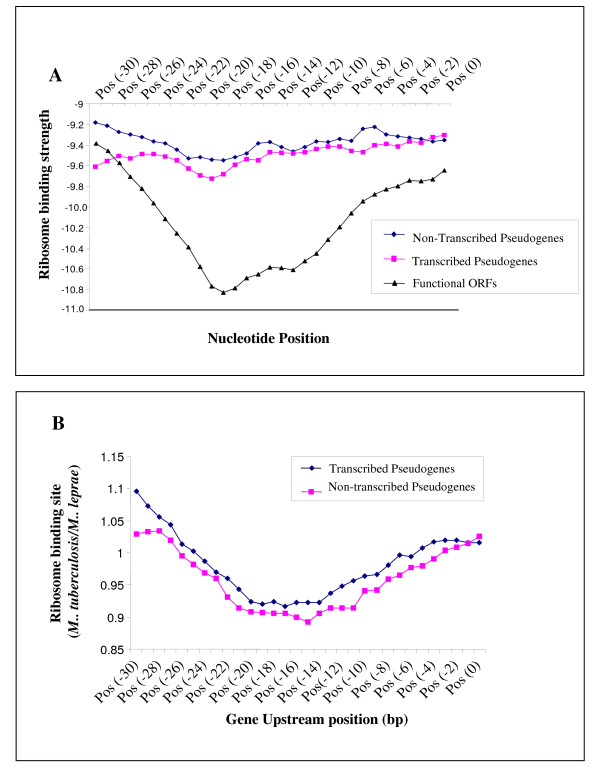
**Ribosome binding strength of *M leprae *genes**. Graphs show the relative ribosomal binding strength of *M. leprae *genes. Panel A shows free energy values of the binding between the 3' sequence of the 16S rRNA and the region upstream of pseudogenes and ORFs. The position of the start codon of the genes is denoted by Pos (0). Lower values indicate stronger binding strength. Panel B shows the RBS ratios of *M. leprae *pseudogenes and their corresponding *M. tuberculosis *homologs.

**Figure 7:**
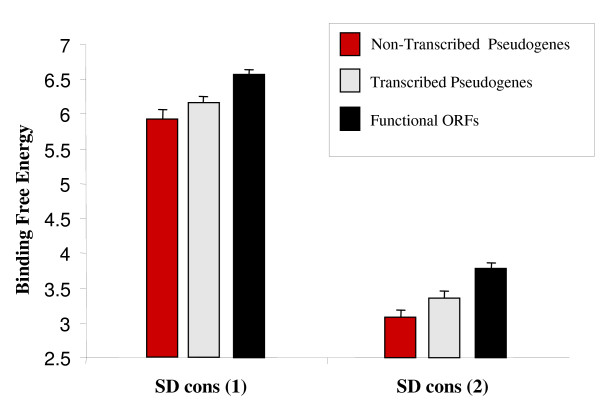
**Mean Shine-Dalgarno (SD) conservation for individual *M. leprae *genes**. This graph shows the SD sequence conservation measured for individual *M. leprae *genes by calculating the difference between the minimum and maximum ribosome binding strength along the 30 bp upstream of gene start, SD (con 1), and by calculating the difference between the minimum ribosome binding strength along the 30 bp and the value at position zero, SD (con 2). Data indicate Means ± S.E for each gene category.

Our data indicated that although the SD sequences of transcribed pseudogenes were somewhat stronger than those of non-transcribed pseudogenes, the ribosome-binding capacity appeared significantly reduced when compared with functional genes (Fig. 6A). In addition, when the SD sequence conservation was estimated in the corresponding *M. tuberculosis *functional orthologs (n = 553), a larger SD degradation is found in *M. leprae*'s non-transcribed pseudogenes relative to transcribed ones (Fig. 6B). However, it was noted that the peak in ribosomal binding efficiency varied along the upstream sequence of the pseudogene, flattening the obtained curve if only mean values were considered. Thus, individual measures of SD sequence conservation were obtained using Methods 1 and 2 described in Materials and Methods and indicated that 38% of transcribed pseudogenes had a putatively conserved SD region (Fig. 7). When the values of SD sequence conservation were compared among the different ORFs, transcribed pseudogenes showed intermediate values between non-transcribed pseudogenes and functional ORFs (Fig. 7; also see Additional File 5 for specific ribosome-binding values for all *M. leprae *genes and pseudogenes). Specifically, genes with degraded SD regions were more common among non-transcribed pseudogenes, whereas the opposite trend was found among ORFs with conserved SD regions (Additional File 5). The values of SD binding strength were always stronger for transcribed pseudogenes at all positions although they are not statistically significant. Thus, although transcribed pseudogenes have a reduced capacity for binding the 16S rRNA, a proportion of them appears to have conserved SD regions and might produce a protein product.

### In frame stop codons within transcribed pseudogenes

**Figure 8:**
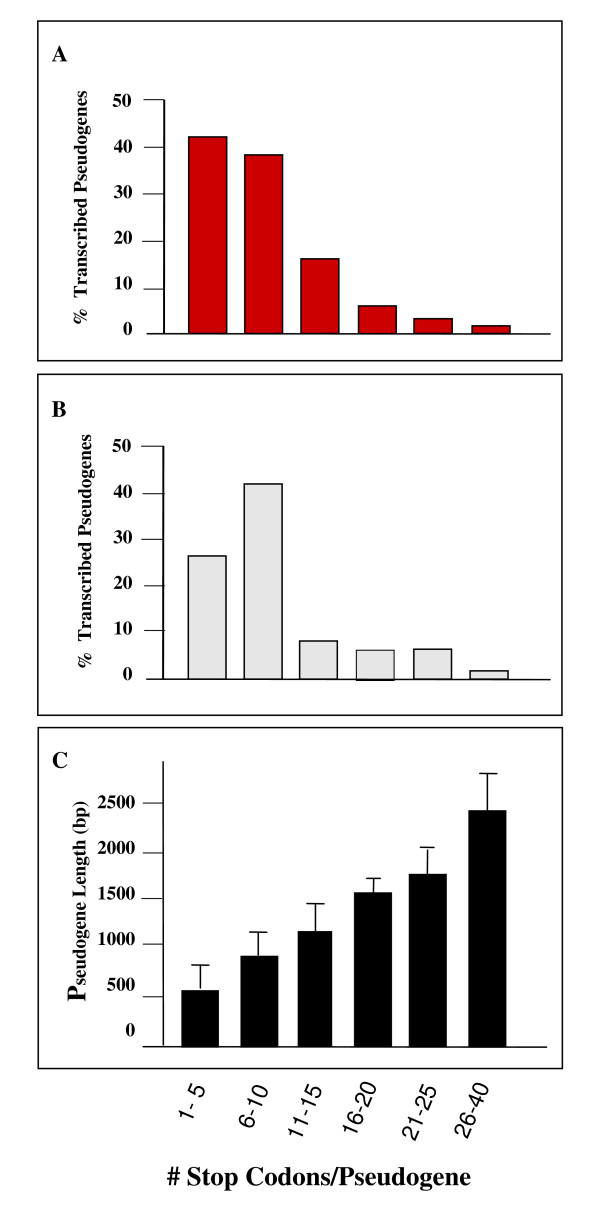
**Translational stop codons within transcribed *M. leprae *pseudogenes**. These graphs show *M. leprae *transcribed pseudogenes containing translational stop codons (UAA, UGA, or UAG). The number of in-frame (5'3' frame 1) stop codons/pseudogene was identified in silico by translation of the DNA sequence for each pseudogene . using (ExPasy Translate Tool: ): Panel A shows the % of transcribed pseudogenes containing the specified number of stop codons/pseudogene (e.g. 1–5, 6–10, etc), obtained by dividing the number of transcribed pseudogenes within each group by that of the total number of transcribed pseudogenes with stop codons; Panel B shows the % transcribed pseudogenes in each group containing translational start codons, obtained by dividing the number of transcribed pseudogenes within each group containing translational start codons by that of the total # pseudogenes with stop codons and translational started codons; Panel C, shows the # of stop codons/group as a function of gene length in base pairs (bp). The mean and standard deviation of the of the gene length (bp) from each group were compared to that of the other groups using GraphPad InStat software and all groups were significantly different (*p *< 0.001) from all other groups except for 21–25 vs 26–40.

Deduced amino acid sequences for all transcribed pseudogenes were obtained using *in silico *translation tools. Results demonstrated that a total of 3,625 in-frame (5'3'frame 1) stop codons were found in 461/486 (95%) of the total number of transcribed pseudogenes and the remaining 25 pseudogenes did not contain stop codons (Additional File 4). The range of transcribed pseudogenes containing stop codons was from 1 to 40 stop codons/pseudogene) with 67% of these transcribed pseudogenes containing at least 5 in-frame stop codons (Fig. 8A). Evaluation of transcribed pseudogenes with translational start codons demonstrated that 95% of these genes contained in-frame stop codons with a range of 1–28/gene (Additional Table 4; Fig. 8B). When the number of stop codons per pseudogene was compared to pseudogene length in base pairs (bp), a significant correlation (*p *< 0.001) was found between gene length and the number of stop codons/pseudogene. For the majority of the expressed pseudogenes, the longer the gene, the greater the number of stop codons (Fig. 8C). Further analysis using the *M. tuberculosis *H37Rv homolog as an estimate of normal frame equivalency for 92 transcribed pseudogenes, demonstrated that deletion mutations have resulted in a reduced coding capacity between 97.7–0.46% of the predicted full-length protein (Additional File 3). This has resulted in an overall loss of 35.4% coding sequence in these pseudogenes. These deletions appeared to have contributed heavily to the presence of the large numbers of stop codons found in these genes and are a dominant force of pseudogene formation in this pathogen.

### Functionality predictions for transcribed pseudogenes

**Figure 9:**
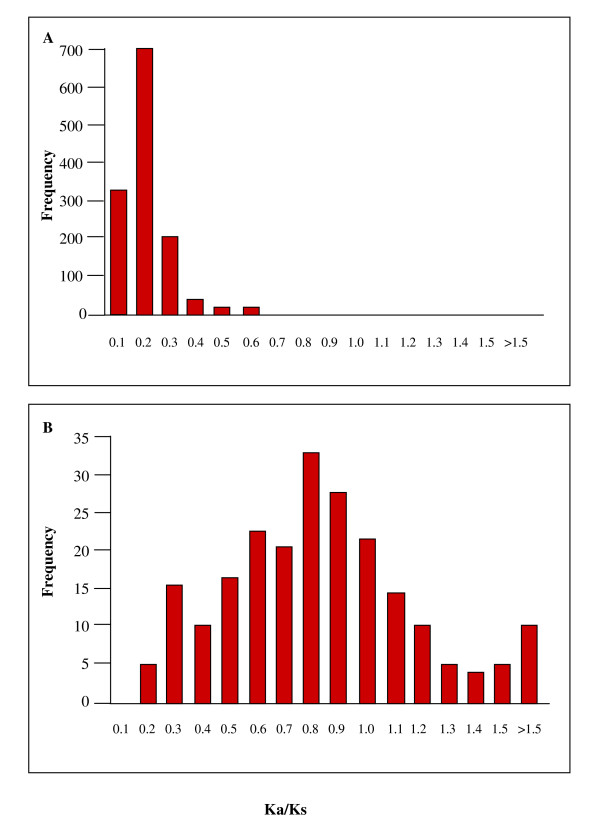
**Functionality of *M. leprae *genes with a functional homolog in *M. tuberculosis *using Ka/Ks ratio analysis**. This graph contains frequency histograms of ratios of nonsynomous (Ka) vs. synonymous (Ks) nucleic acid substitutions of *M. leprae *genes when compared to their homologous genes in *M. tuberculosis*. Panel A represents the frequency histogram for Ka/Ks ratios of *M. leprae *functional ORFs. Panel B represents the frequency histogram for Ka/Ks ratios of *M. leprae *pseudogenes that have a functional homolog in *M. tuberculosis *H37Rv.

**Figure 10:**
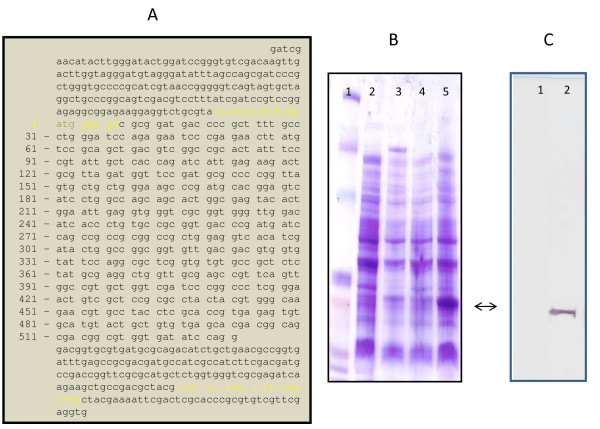
**Cloning and protein expression of the *pyrR *pseudogene in *E. coli***. Crude cell lysates of recombinant BL21-Star *E. coli *cells containing the *M. leprae pyrR *pseudogene (*E. coli*::pET200/D/TOPO/ML*pyrR*) were separated on 4–20% SDS-PAGE gradient gels, and the histidine epitope was detected by Western blotting using the Anti-Xpress™ antibody: Panel A, shows the *M. leprae pyrR *pseudogene sequence for PCR amplification, yellow bases depict primers for PCR, the numbered sequences are the coding region; Panel B, is an SDS-PAGE gel stained with commassie brilliant blue, containing proteins from the recombinant *E. coli *strains: Lane 1, Kaleidoscope Prestained Standards Invitrogen #161-0324; Lane 2, *E. coli*::pET200/D/lacZ; Lane 3, *E. coli*::pET200/D/lacZ, IPTG-induced 18 hr; Lane 4, *E. coli*::pET200/D-TOPO/ML*pyrR*; Lane 5, *E. coli*::pET200/D-TOPO/ML*pyrR*, IPTG-induced 18 hr. Panel C represents a Western blot for the histidine epitope on the recombinant *E. coli*::pET200/D-TOPO/ML*pyrR *using crude cell lysates from IPTG-induced 18 hr samples: Lane 1, *E. coli*::pET200/D/lacZ; Lane2, *E. coli*::pET200/D/TOPO/ML*pyrR*.

When *M. leprae *genes were analyzed for potential functionality by using homologs in *M. tuberculosis *H37Rv and Ka/Ks ratio analysis, results demonstrated that ORFs, annotated as functional, contained a mean Ka/Ks ratio of 0.31 ± 0.16 (Fig. 9A). Ninety percent of ORFs with Ka/Ks values > 0.5 were identified as hypothetical proteins or ribosomal proteins (data not shown). In comparison, of 216 pseudogenes analyzed (i.e. those with a functional homolog in *M. tuberculosis *for which unambiguous alignments could be obtained) the mean Ka/Ks ratio was 0.78 ± 0.35 (Fig. 9B). Thus, pseudogenes have undergone a dramatic shift in their Ka/Ks ratios, indicating they accumulate replacement substitutions at higher rates than functional genes. Given that Ka/Ks ratios of 1 cannot be achieved unless the *M. tuberculosis *ortholog used for comparison is also a pseudogene, the observed Ka/Ks values are extremely high and suggest that most pseudogenes evolve under lack of selection. In fact, only 48/216 (22%) of pseudogenes contained Ka/Ks ratios ≤ 0.5, indicating that they could potentially encode a functional protein. But even these cases could correspond to neutrally-evolving ORFs that have lost functionality only recently. However, of these 48 pseudogenes with low Ka/Ks ratios, transcripts were detected for only 23. When one of these pseudogenes, *pyrR *(Ka/Ks ratio = 0.09) containing a strong SD, a functional promoter, and a translational start codon (ATG) was analyzed for the ability to produce a translational product in *E. coli*, a protein product was observed (Fig. 10).

Thus, most pseudogenes, including those that are transcribed, appear to be under very low selection strength. It is also worth mentioning that 15/216 (7%) have Ka/Ks ratios > 1.4 (Fig. 8B), whereas these cases are basically absent in functional genes. These higher-than-expected non-synonymous substitutions are characteristic of proteins under positive selection, but only 4 of them are transcribed, and therefore, it is unlikely that these high ratios indicate accelerated protein evolution but simply sequence deterioration. These analyses could be considerably improved when the genome of the second strain of *M. leprae *BR4923 (just recently available) is analyzed, so that sequence evolutionary patterns could be compared between pseudogenes in both strains.

## Discussion

Pseudogenes are considered disabled copies of functional genes that were once active in the ancient genome and their identification has been relatively rare until the recent availability of a large number of fully sequenced and annotated genomes and the improvement in detection algorithms [17-19]. Analysis of these genomes demonstrated that pseudogenes are much more common than previously thought and that pseudogenes can represent a significant fraction of the genome [[5,18,19]; ]. As a result, the coding potential of genomes has been shown to be substantially lower than originally predicted. For example, the human genome contains 16,326 pseudogenes and *Escherichia coli *K-12 genome, once thought to only possess a few pseudogenes, has been shown to harbor 134 inactivated genes. Mycobacterial species are no exception. *M. tuberculosis *H37Rv contains 278 inactivated genes  and the recently sequenced *M. ulcerans *genome has 727 pseudogenes (BuruList Web Server: ) [20]. The case of pseudogenes in *M. leprae *is very dramatic with over 1100 being documented [4,5,9]. This represents the largest number of any bacterial genome sequenced to date. These data strongly suggest that genome down-sizing through the accumulation of pseudogenes, as well as gene loss, has resulted in the very specialized requirements for *M. leprae *growth.

Although the precise mechanism resulting in the formation of this large number of pseudogenes in *M. leprae *is unclear, several possible mechanisms have been defined. It has been suggested that the loss of dnaQ-mediated proofreading activities of the DNA polymerase III and large-scale rearrangements and deletions arising from homologous recombination events may have contributed to this accumulation of pseudogenes [4,5]. The loss of sigma factors [21] and two-component systems [22] have also been proposed as possible mechanisms in *M. leprae *pseudogenization. The dynamics of this reductive process in *M. leprae *has recently been studied by reconstructing the gene content of the last common ancestor of *M. leprae *and its closest relative *M. tuberculosis *and comparing it with the present *M. leprae *genome [23]. Data from this study suggest that the loss of ancestral genes resulted in the loss of functional genes of *M. leprae's *ancestor and its divergence from *M. tuberculosis *and that pseudogenization events appear to be recent gradual evolutionary events in *M. leprae's *lineage (within the last 20 million years).

Pseudogene accumulation might promote adaptive microevolution resulting in transitioning from a free-living to a mutualistic lifestyle [1,2]; from multiple hosts to specific hosts and ultimately specific host cells. Therefore, pseudogenization of *M. leprae's *sigma factors [19] and stress response genes, resulting in limited response to environmental stress conditions [24], may have contributed at least in part to its adaptive evolution and to its extremely specialized niche within peripheral macrophages [24,25] and Schwann cells of peripheral nerves in humans [26].

In general, pseudogenes are considered to be 'junk' DNA sequences that are in the process of being removed from the genome. However, recently we and others have demonstrated the presence of a small number of pseudogene transcripts in *M. leprae *[11,12] and other bacterial species [27,28]. In addition, others have found that transcribed pseudogenes can be functional [29].

In the present study, further characterization of the overall pseudogene transcriptional profile of *M. leprae *in the nu/*nu *mouse foot pad granulomatous tissue by global DNA array and RT-PCR analyses demonstrated that not only does *M. leprae *possess the highest number of pseudogenes/genome it also possesses the highest rate of bacterial pseudogene transcription documented to date. There was no apparent bias for transcription of pseudogenes in *M. leprae *based on chromosomal location or functional gene category. Although the highest percentage of transcribed pseudogenes was found in functional category V (hypothetical proteins), this finding was not surprising as this category contains the largest percentage of pseudogenes in the genome [5]. Many pseudogenes belong to gene families that are large in close relatives such as *M. tuberculosis *but are simplified during the loss of redundancy that takes place after niche specialization [8]. Results of the present study demonstrated that a large number of these degenerated ORFs, which may no longer code for their appropriate functions, were expressed in *M. leprae *using transcriptional machinery, metabolic resources and energy without potential benefit to this organism. These direct and indirect costs have previously been suggested to select against the expression of pseudogenes in *M. leprae *by the erosion of sequences involved in transcription initiation [4]. Therefore, even though a large number of *M. leprae *pseudogenes are transcriptionally active, approximately 60% of *M. leprae's *pseudogenes are transcriptionally silent, presumably by this or similar mechanisms.

*In silico *analysis of transcribed pseudogenes suggested potential mechanisms for their transcription. Their positioning within gene clusters (operon-like organizations), or downstream of transcribed ORFs, along with the paucity of intrinsic terminators between functional ORFs and transcribed pseudogenes implies that several pseudogenes are transcribed via a read-through manner. These data support a previous study which demonstrated that ~74% of *M. leprae *ORFs lacked detectable intrinsic transcriptional terminators [16]. An exception to this was found in the present study when the transcriptional pair ML0180c-ML0179c (pseudogene), containing a strong terminator sequence (ΔG -38.4) within the ML0180c coding region, was found to be transcribed as a single gene transcript product. The question is why is the terminator not functional? Previous work by our group has shown that terminators do not function if they are inside coding regions. There could be various reasons for this, prominently the presence of ribosomes or formation of antitermination complexes. In this case, the terminator is inside the pseudogene coding region and factor(s) which prevent termination functions inside coding regions could come into play. Sequences upstream and downstream of terminators have also been shown to be important in some cases. These could be the reason(s) for its lack of functioning. Also it must be noted that ΔG is an important, but not the sole indicator of terminator efficiency. In fact, our work has also shown that most terminators in *M. leprae *have a ΔG lower than this value.

The present study also demonstrated that *rho *(ML1132) and *ndk *(ML1469c), a nucleoside diphosphate kinase associated with its activity [30], were among the 1353 genes expressed. However, to date nothing is known about *rho*-dependent transcript termination in *M. leprae *and therefore, the significance of this for pseudogene gene expression is unknown. In addition co-transcription of genes of unrelated function has been shown in intracellular species that have undergone massive genome reduction and low selection strength such as *Buchnera*, where after the elimination of DNA segments that included promoter regions, two unrelated genes ended up physically linked [31] and were shown by microarray analysis to be co-transcribed [32]. Thus, these imperfect regulatory mechanisms in which promoter-less ORFs or pseudogenes are unnecessarily expressed may not be uncommon in species undergoing low selection strength, such as those under episodes of genetic drift and small population sizes.

However, not all *M. leprae *pseudogenes appear to rely on read-through transcription as a mechanism of transcription. Putative promoters were identified in silico in the upstream region of *M. leprae *pseudogenes. When 10 of these were tested for promoter activity in a promoterless reporter *E. coli *system, all were positive. Therefore, while the selection against the expression of pseudogenes in *M. leprae *by the erosion of sequences involved in transcription initiation appears to be an effective transcriptional mechanism for "silencing" *M. leprae *pseudogenes, the presence of functional promoters contributes to pseudogene transcription in *M. leprae*.

Prokaryotic mRNAs generally contain within their 5'-UTRs an SD sequence that serves as a ribosome-binding site [33]. The loss of functional SD sequences results in the lack of efficient translational capability and therefore results in a reduction or loss of protein production. Recently it has been reported that the SD sequences of *M. leprae *pseudogenes are highly degraded or degenerate suggesting that translation is impaired in nonfunctional open reading frames (pseudogenes) in this pathogen and that this potentially reduces the metabolic investment on faulty proteins because, although pseudogenes can persist for long time periods in the genome, they would be effectively "silenced" [4]. The present study confirmed these results and further demonstrated that although they have lower ribosomal binding strength than ORFs, transcribed pseudogenes have higher ribosomal binding strength than non-transcribed pseudogenes. Therefore these data strongly suggest that some transcribed pseudogenes are actually translated in *M. leprae*. To test this hypothesis, the promoter, SD (strong ribosomal binding strength), start codon and partial coding region of the *pyrR *(ML0531) pseudogene was fused into the *gfp *gene in a promoterless reporter plasmid lacking a SD site and was transformed into *E. coli*. Results of this preliminary experiment suggested that the *pyrR *SD site initiated ribosomal binding and resulted in the translation of the *pyrR*-*gfp *fusion protein product yielding the green fluorescent phenotype. Thus, although the results of this study indicate that most pseudogenes have either no recognizable SD or weak SD sequences for binding to the anti-SD sequence of the 3' region of the 16S rRNA, some of the transcribed pseudogenes have intact ribosome-binding sequences of similar strength to the orthologs in *M. tuberculosis*.

In addition, the current study demonstrated that the majority of transcribed pseudogenes lack traditional prokaryotic translational start codons. It has been shown that alteration of start codons results in loss of translational efficiency [32,33]. Even though the lack of these sequences in the majority of *M. leprae *pseudogene transcripts appears to be an effective mechanism for translational "silencing", to date this has not yet been experimentally confirmed.

In-frame stop codons (elementary property that distinguishes a pseudogene from a functioning gene) were present in 95% of transcribed pseudogenes, whether or not they contained start codons. Therefore, if translation of transcribed pseudogenes initiates, a truncated protein product should result from the majority of *M. leprae *pseudogenes. In rare instances, the protein fragment is still functional as bad codons can also be bypassed or edited at the level of mRNA by recoding mechanisms. Recoding is the reprogramming of mRNA translation by localized alterations in the standard translational rules and recoding products can play critical cellular roles [34]. Typically three classes of recoding are known: 1) frameshift recoding; 2) bypass (hopping) recoding; and 3) codon redefinition involving site-specific recognition (usually but not limited to stop codon). Recoding is utilized in the expression of a minority of genes in probably all organisms and has been documented in *M. avium*, [Selenocysteine incorporation at the stop codon (UGA) to yield formate dehydrogenase ]. To date recoding has not been documented in *M. leprae *or its close relative *M. tuberculosis*. However, if recoding does occur in *M. leprae*, it is unlikely that transcripts would be recoded to yield full length sequences when multiple stop codons occur in a single coding sequence. It is estimated that 80% of transcribed pseudogenes contain at least 3 stop codons within their sequence and 90% of these pseudogenes have < 50% of the predicted full-length protein when compared to the *M. tuberculosis *homolog due to deletion mutations. Therefore, it is predicted that if translated, these sequences will result in truncated proteins.

Using the non-synonymous to synonymous substitutions analysis as a measure of potential functionality of pseudogenes, we showed that only one third of these genes had similar Ka/Ks ratios to functional genes, regardless of whether they are transcribed or not. As explained above, this is an upper limit because part of the analyzed sequence evolution corresponds to the *M. tuberculosis *functional orthologs and because the pseudogenization process could be recent for some genes and therefore their Ka/Ks ratios would be close to normal. Therefore, although the number of pseudogenes for which unambiguous Ka/Ks ratios could be obtained was small, and at least one of these with a low Ka/Ks ratio was translated, these data suggest that most transcribed pseudogenes are in the process of degradation. However, this is an upper estimate because of the potential short time passed after pseudogenization and because part of the substitutions correspond to the functional homolog in *M tuberculosis *taken as reference. Additional support for these conclusions is that even though protein expression data has demonstrated the presence of > 300 proteins in protein extracts from armadillo-derived *M. leprae*, no pseudogene products were identified [14,15].

## Conclusion

The data presented in this study strongly suggest that even though a large number of *M. leprae's *pseudogenes are transcriptionally active, translational "silencing" mechanisms ensure that valuable metabolites and energy are not wasted to produce proteins from the majority of these transcripts which have no apparent benefit for cellular survival or growth of *M. leprae*. However, it is unclear whether these pseudogene transcripts have an additional detrimental effect on *M. leprae*. Nevertheless, some pseudogene transcripts do appear to be capable of producing protein products. These genes and their potential translational products need to be studied more extensively to understand their full biological impact on *M. leprae*.

## Methods

### RNA purification and cDNA production

*M. leprae *was purified from the granulomatous hind footpad tissue of four individual nu/*nu *nude mice, six months post-infection as previously described [35]. *M. leprae *RNA was purified from individual bacterial preparations as previously described using TRIzol^® ^(Invitrogen, Carlsbad, CA), FastPrep Blue RNA tubes and mechanical extraction using a FastPrep^® ^120 Instrument [36]. DNA was removed from these preparations using the Turbo DNA-free™ kit (Ambion, Austin, TX). DNA-free RNA aliquots were then stored at -80°C. This RNA was used for DNA microarray analysis or converted to cDNA for RT-PCR analysis using 1 μg RNA and the Advantage RT-for-PCR Kit (BD BioSciences, Clontech, Mountain View, CA) using random hexamers. Controls for DNA contamination consisted of 1 μg RNA incubated with the reverse transcription reagents, excluding the reverse transcriptase (RT-). Template cDNA was also made from BALB/c mouse spleen total RNA (BD Biosciences, Clontech).

### M. leprae gene expression by microarray analysis

*M. leprae *whole genome DNA microarrays representing the 1,614 annotated ORFs and 1,133 identified pseudogenes, were obtained from the Leprosy Research Support and Maintenance of an Armadillo Colony Post-Genome Era, Part I: Leprosy Research Support Contract (NO1 AI-25469) at Colorado State University. Microarray experiments were performed using previously described protocols [37]. Microarrays were scanned using a Bio-Rad VersArray Chip Reader (Bio-Rad, Carlsbad, CA) and using SpotFinder Software (manufacturer) to quantify fluorescence. Genes were positive for transcription if the average mean signal to noise ratios (SNR) were > 2-fold for samples analyzed. Transcribed pseudogenes were then mapped to the *M. leprae *chromosome and functional gene categories which have transcribed pseudogenes were identified .

### RT-PCR amplification

To validate ~20% of genes positive by microarray analysis, RT-PCR was performed using Thai-53 *M. leprae *cDNA, primers for PCR amplification [based on gene sequences from the *M. leprae *TN genome / using PrimerQuest ], and PCR analysis using recommended primer annealing temperature and 40 cycles of PCR. *M. leprae *DNA (1 ng) was used as a positive control. Reactions without reverse transcriptase (RT (-) reactions), buffer and mouse cDNA were used as negative controls for each assay. Amplicons were observed in ethidium bromide-stained 2% agarose gels using Gel Doc 2000 Gel Documentation System (Bio-Rad) and the amplicon sequence was confirmed using automated DNA sequencing.

### Identification of transcriptional read-through mechanisms

To identify *M. leprae *pseudogenes potentially transcribed as a result of read-through transcription, the presence of these pseudogenes within gene clusters or directly downstream of transcribed ORFs was analyzed using Gene Cluster analysis (GeneChords-) and the *M. leprae *TN mapping data . *M. leprae *cDNA was used as the template to amplify fragments representing the read-trough products of predicted size using PCR and forward primers within the upstream ORFs and reverse primers within the pseudogenes or in the genes downstream of the pseudogene (Fig. 3A). Products were analyzed for their predicted fragment sizes using agarose gel electrophoresis and the DNA sequence of the resultant PCR amplicons was confirmed by automated DNA sequencing.

### Identification of stem loop structures indicative of intrinsic termination sequences

Since read-through transcription relies on the absence of transcript termination structures between the 3'-UTRs of transcribed ORFs and downstream genes or pseudogenes, the presence of stem loop structures, indicative of intrinsic terminators, was investigated in the annotated genome of *M. leprae *TN strain using the algorithm Genome Scanner for Terminators (GeSTer) [16,38]. The program accepts whole genome sequence information from GenBank (NCBI), and searches the sequences -20 to +270 with respect to the stop codon in the 3'-UTR of upstream ORFs for palindromic sequences, which could potentially form stem-loop structures when transcribed. It then sorts out the structures based on their ΔG. GeSTer further defines a genomic ΔG_cutoff _which is a function of the genomic G+C content of the bacterial species. Palindromic structures with ΔG value more negative than this cut-off are only considered as potential intrinsic terminators.

### In silico identification of putative pseudogene promoters

Independent pseudogene transcription requires the presence of functional a promoter in the (5'UTR) of these genes. Promoters need DNA bend regions as the RNA polymerase complex initiates strand separation at the promoter -10 regions [39]. Putative pseudogene promoters were located by DNA curvature (bend) analysis [40,41] with the "bend-it" server  using DNase I parameters [42] and the consensus bendability scale [42] with a 31 size sliding window and simple smoothing of plots. Upstream 200–300 nucleotide plots of intrinsic curvature, bendability, complexity and GC content troughs were used to locate promoter regions through coincidence of peaks and troughs. Intrinsic curvature peak heights of less than 5 degrees per helical turn were discarded [42]. The *sigA *promoter region 38 was used as a standard [43]. When these peaks and troughs coincided, the region was assigned a putative promoter site. To avoid pseudogenization effects on upstream regions it was necessary to use ClustalW alignments with upstream regions from normal mycobacterial genes of the Mtb complex, the MMAR-MUL and MAV-MAP complexes. If alignments could not be made, the ML data was discarded. ClustalW  cross-species mycobacterial DNA alignments were used to assign putative promoter -10, -35 elements, initiation sites (I) and start codons to the predicted promoter-like regions and determine the distance (bp) of the promoter upstream from the start codon.

### In vitro promoter analysis

To further characterize pseudogene promoters previously identified by "bend-it" DNA curvature and alignment analyses, approximately 150 bp fragments of upstream sequence of 10 pseudogenes containing putative promoter regions were amplified from *M. leprae *Thai-53 DNA using standard PCR, primers (Additional File 6) and cloned into the *p*Glow-TOPO-TA vector which contains a promoterless Cycle 3 GFP mutant reporter from the *p*Glow-TOPO-TA-Expression-Kit (Invitrogen). *E. coli *XL1-Blue Supercompetent cells were then transformed with these plasmids. Fluorescence was analyzed from ampicillin-resistant (100 μg/ml ampicillin in Luria Bertani agar) bacterial clones by mixing a portion of each colony with 10 μl sterile PBS and placing the bacterial suspension onto a glass slide. A cover slip was applied and slides were examined using a Nikon Eclipse E400 fluorescent microscope using a FITC/gfp filter (excitation/emissionmaxima of 480 nm/560 nm). Positive clones were considered those that contained fluorescence levels above cultures that contained only the *p*Glow-TOPO-TA re-circularized vector.

### Prediction of pseudogene translational potential

The translational potential for transcribed pseudogenes was determined *in silico *by analysis of start codons (AUG, GUG, and UUG) at the 5' end of predicted pseudogenes using the *M. leprae *TN strain genome sequence . Since the ability of a transcript to be translated appears to be dependent on conservation of SD sequences which bind the transcript to its complementary sequence in the 3'region of the 16S rRNA, upstream regions of these pseudogenes were examined for SD binding strength using previously described protocols [44]. These procedures calculate, based on base pair formation rules [45], the free energy values for the binding of the 3' end of the 16S rRNA with the region preceding each ORF at different positions (position zero indicates gene start). Lower free-energy values indicate higher binding strength. Estimates of SD sequence conservation for individual ORFs were obtained by quantifying the difference between the maximum and minimum free energy along the 50 nucleotides preceding the start of the gene (Method 1) and the difference between the free-energy value at position zero and the minimum value along the preceding 50 nucleotides (Method 2). Based on SD sequence conservation in *M. tuberculosis*, which has not undergone a pseudogenization process, values > 7 and 5 were taken as indicative of a conserved, functional SD region by the first and second measure, respectively.

### Prediction of pseudogene functionality

The translational potential for transcribed pseudogenes was also investigated by estimating selection strength by calculating the rates of synonymous (Ks) and nonsynonomous (Ka) DNA substitutions (Ka/Ks ratios) in these sequences and compared to their corresponding functional homologs in *M. tuberculosis *H37Rv. Sequences of *M. leprae *pseudogenes and their corresponding *M. tuberculosis *H37Rv functional homologs were aligned using the program Pileup of the GCG Wisconsin package (Genetics Computer Group 1997). Only unambiguous alignments were considered. Rates of synonymous and non-synonymous substitutions were calculated using the Diverge command in GCG that applies Li's algorithm [46] with modifications [47]. The Ka/Ks ratio is indicative of selection strength: in functional genes, synonymous substitutions are more frequent than replacement substitutions. Nevertheless, the mean ratio was also calculated for *M. leprae *functional ORFs for comparison to pseudogenes. Typically in other bacteria if the Ka/Ks ratio is around 1, substitutions are equally frequent at all three codon positions and the ORF is likely to be a pseudogene. However, only 216 pseudogenes could be analyzed because of lack of homology with *M. tuberculosis*, alignment ambiguity, or because the start codon and/or reading frame could not be unequivocally identified.

A subset of transcriptionally active pseudogenes with translational start codons were further analyzed to determine the percentage of the predicted full-length protein of pseudogene when compared to the *M. tuberculosis *H37Rv homolog (% Rv) using the deduced translated protein sequences for *M. leprae *ExPasy Translate Tool  and ClustalW alignment  to the *M. tuberculosis *homolog.

### Cloning and expression of the pyrR pseudogene protein

Primers designed to amplify the coding region corresponding to the entire 532 bp coding region of the *pyrR *pseudogene (including stop codons at codon 158 and 166 and beyond the end of the coding sequence) from Thai-53 DNA and containing CACC on the 5'terminus of the forward primer (Fig. 10A) were used with standard PCR and directionally cloned into the linearized, topoisomerase I-activated Champion™ pET200/D-TOPO vector (Invitrogen), containing an N-terminus 6× His-tag, an N-terminal tag of a β-galactosidase and T7*lac *promoter for high-level expression, and transformed into One Shot^® ^BL21 Star™ (DE3) and Top10 for storage (Invitrogen) according to manufacturer's recommendations. A kanamycin-resistant (50 μg/ml in LB agar) clone BL21 Star™ was verified to contain the *pyrR *insert by PCR and subsequent automated DNA sequencing (*E. coli::*pET200/D-TOPO/ML*pyrR*). A culture was grown to an OD_600 _= 0.6 and induced for several hours with a final concentration of 1 mM isopropyl β-D thiogalactopyranoside (IPTG) (Sigma-Aldrich, St. Louis, MO). Crude cell lysates from both non-induced and induced *E. coli::*pET200/D-TOPO/ML*pyrR *after 18 hr incubation at 37°C were separated using a SDS-PAGE (4% to 20% discontinuous gradient polyacrylamide gel, stained with coomassie brilliant blue (Bio-Rad), and compared against Kaleidoscope Pre-stained Standard markers (Bio-Rad). A Western blot using the Anti-Xpress™ antibody (Invitrogen) was used to verify the presence of the polyhistidine epitope on the recombinant protein product. Crude cell lysates from *E. coli::*pET200/D-TOPO/lacZ (Champion™ pET200/D-TOPO vector Kit), treated under the same conditions described above, were used as a control for this experiment however, this control lacked the histidine epitope tag.

### Statistical analysis of data

Statistical analysis of data for this study was obtained from the comparison of means and standard deviations of raw data and performed using One-way Analysis of Variance (ANOVA) using Tukey-Kramer Multiple Comparisons Test GraphPad InStat software. All data with *p *< 0.05 were considered significant.

## Authors' contributions

DLW: Project manager, experimental design, bioinformatics, manuscript writing and editing. TLP and ANM: Bioinformatics, cloning and promoter analysis, RNA purification, RT-PCR validation experiments and cloning and expression of PyrR protein. RS and AA*: *M. leprae *DNA microarray experiments and analysis, and assistance with writing and editing manuscript. VN and AM: Intrinsic terminationexperiments and assistance with writing and editing manuscript. AM: SD and Ka/Ks analyses and assistance with writing and editing manuscript. NM: Promoter "bend-it" and promoter alignment analysis and assistance with writing and editing manuscript. MM: Assistance with editing manuscript. TPG: Assistance with protein expression, experimental design and editing manuscript.

*Passed away before completion of the manuscript.

## Supplementary Material

Additional file 1 **Transcriptional profile of *M. leprae *Thai-53 from the granulomatous foot pad tissue of nu/*nu *mouse using DNA microarray and RT-PCR analysis**. The *M. leprae *gene transcripts detected using DNA microarray analysis experiments were performed using previously described protocols [37]. Microarrays were scanned using Bio-Rad VersArray Chip Reader and SpotFinder software to quantify fluorescence. Genes were positive for transcription if signal-to-noise ratios (SNR) were > 2-fold (raw data was deposited the public repository NCBI's Gene Expression Omnibus (GEO and are accessible through GEO Series accession number GSE17191 study at: . In addition, some gene transcripts were detected using RT-PCR and some of which have been previously published [11]. ^1^*Mycobacterium leprae *gene #: . ^2^*Mycobacterium tuberculosis *gene #: . ^3^Gene List: . ^4^*M. leprae *pseudogenes which have tested positive using for *M. leprae *gene transcripts using reverse transcriptase-PCR and cDNA from nude mouse footpad-derived *M. leprae *Thai-53. ^5^Data used with permission from Patrick Brennan [14,15]. Click here for file

Additional file 2 **Intrinsic terminators in *M. leprae***. This table gives predicted intrinsic terminators in the upstream region of *M. leprae *genes as a function of the genomic G+C content and palindrome-like structures with ΔG < -14 are considered as potential intrinsic terminators [16]. ^1^*Mycobacterium leprae *gene #: . ^2^Algorithm Genome Scanner for Terminators (GeSTer) [16,38]. Click here for file

Additional file 3 ***M. leprae *pseudogene promoter-like regions and truncation of transcribed pseudogenes compared to *M. tuberculosis *homologs**. Using the *M. leprae *sequence data from 92 transcribed pseudogenes , the presence of promoter-like regions and truncation of transcribed pseudogenes were analyzed and compared to *M. tuberculosis *homologs using "bend-it" DNA curvature analysis . ML#: . Rv#: . Start: Start codon as assigned to pseudogene . ISp bp: Upstream intergenic space in bp as assigned . HP deg/tn: Highest DNA curvature peak above threshold in degrees/helical turn found in the intergenic space as assigned by "bend-it" using DNase I values for bend parameters [41]. Pks: Number of static DNA curvature peaks with threshold > 5 degrees in the intergenic space as assigned by "bend-it" using DNase I values for bend parameters. # Stop: Number of stop codons present in the pseudogene frame. % Rv: The percentage of coding frame available before the first stop codon compared to the *Mycobacterium tuberculosis *ortholog as assigned in . Click here for file

Additional file 4 **Bioinformatics of 486 transcribed pseudogenes of *M. leprae***. Using *M. leprae *sequence data , translational start codons (ATG, GTG, TTG) and translational stop codons (UGA, UAA, UAG) are listed as obtained using 5' 3' Frame 1 data from ExPasy Translate Tool  for each transcribed pseudogene: ^1 ^*Mycobacterium leprae *gene #: . ^2 ^*Mycobacterium tuberculosis *gene #: . ^3^% Rv = % of the predicted full-length protein (in silico translated) when compared to the *M. tuberculosis *H37Rv homolog. Click here for file

Additional file 5 **Shine-Dalgarno signal strength of *M. leprae *genes**. The translational potential for transcribed pseudogenes was determined by analysis of start codons (AUG, GUG, and UUG) at the 5' end of predicted pseudogenes using the *M. leprae *TN strain genome sequence ^1^ and regions upstream of these pseudogenes were examined for SD binding strength. The free energy values for the binding of the 3' end of the 16S rRNA with the region preceding each ORF at different positions (position zero indicates gene start). Lower free-energy values indicate higher binding strength. Estimates of SD sequence conservation for individual ORFs were obtained by quantifying the difference between the ^2^minimum and ^3^maximum free energy along the 50 nucleotides preceding the start of the gene ^4^(SD cons 1) and the difference between the free-energy value at position zero and the minimum value along the preceding 50 nucleotides ^5^(SD cons 2). Click here for file

Additional file 6 ***M. leprae *pseudogene promoter sequences for *in vitro *functional promoter analysis**. This figure includes the 5'UTRs of *M. leprae *pseudogenes for cloning into the promoterless reporter vector *p*Glow-TOPO-TA promoterless reporter-*gfp *vector for identification of promoter activity. The legend designates which area corresponds to primers for PCR amplification the promoter region including -35 and -10- and initiation site (+1) as well as their position relative to the start codon for the pseudogene. Click here for file

## References

[B1] Tong Z, Zhou D, Song Y, Zhang L, Pei D, Han Y, Pang X, Li M, Cui B, Wang J, Guo Z, Qi Z, Jin L, Zhai J, Du Z, Wang J, Wang X, Yu J, Wang J, Huang P, Yang H, Yang R (2005). Pseudogene accumulation might promote the adaptive microevolution of *Yersinia pestis*. J Med Microbiol.

[B2] Toh H, Weiss BL, Perkin SA, Yamashita A, Oshima K, Hattori M, Aksoy S (2006). Massive genome erosion and functional adaptations provide insights into the symbiotic lifestyle of *Sodalis glossinidius *in the tsetse host. Genome Res.

[B3] Andersson JO, Andersson SG (1999). Genome degradation is an ongoing process in Rickettsia. Mol Biol Evol.

[B4] Mira A, Pushker R (2005). The silencing of pseudogenes. Mol Biol Evol.

[B5] Cole ST, Eiglmeier K, Parkhill J, James KD, Thomson NR, Wheeler PR, Honore N, Garnier T, Churcher C, Harris D, Mungall K, Basham D, Brown D, Chillingworth T, Connor R, Davies RM, Devlin K, Duthoy S, Feltwell T, Fraser A, Hamlin N, Holroyd S, Hornsby T, Jagels K, Lacroix C, Maclean J, Moule S, Murphy L, Oliver K, Quail MA, Rajandream MA, Rutherford KM, Rutter S, Seeger K, Simon S, Simmonds M, Skelton J, Squares R, Squares S, Stevens K, Taylor K, Whitehead S, Woodward JR, Barrell BG (2001). Massive gene decay in the leprosy bacillus. Nature.

[B6] Cole ST, Brosch R, Parkhill J, Garnier T, Churcher C, Harris D, Gordon SV, Eiglmeier K, Gas S, Barry CE, Tekaia F, Badcock K, Basham D, Brown D, Chillingworth T, Connor R, Davies R, Devlin K, Feltwell T, Gentles S, Hamlin N, Holroyd S, Hornsby T, Jagels K, Krogh A, McLean J, Moule S, Murphy L, Oliver K, Osborne J, Quail MA, Rajandream MA, Rogers J, Rutter S, Seeger K, Skelton J, Squares R, Squares S, Sulston JE, Taylor K, Whitehead S, Barrell BG (1998). Deciphering the biology of *Mycobacterium tuberculosis *from the complete genome sequence. Nature.

[B7] Moran NA, Wernegreen JJ (2000). Lifestyle evolution in symbiotic bacteria: insights from genomics. Trends Ecol Evol.

[B8] Rocha EP, Danchin A (2002). Base composition bias might result from competition for metabolic resources. Trends Genet.

[B9] Eiglmeier K, Parkhill J, Honore N, Garnier T, Tekaia F, Telenti A, Klatser P, James KD, Thomson NR, Wheeler PR, Churcher C, Harris D, Mungall K, Barrell BG, Cole ST (2001). The decaying genome of *Mycobacterium leprae*. Lepr Rev.

[B10] Pushker R, Mira A, Rodríguez-Valera F (2004). Comparative genomics of gene-family size in closely related bacteria. Genome Biol.

[B11] Williams DL, Torrero M, Wheeler PR, Truman RW, Yoder M, Morrison N, Bishai WR, Gillis TP (2004). Biological implications of *Mycobacterium leprae *gene expression during infection. J Mol Microbiol Biotechnol.

[B12] Suzuki K, Nakata N, Bang PD, Ishii N, Makino M (2006). High-level expression of pseudogenes in *Mycobacterium leprae*. FEMS Microbiol Lett.

[B13] Mira A, Pushker R, Rodriguez-Valera F (2006). The Neolithic revolution of bacterial genomes. Trends Microbiol.

[B14] Marques MA, Espinosa BJ, Xavier da Silveira EK, Pessolani MC, Chapeaurouge A, Perales J, Dobos KM, Belisle JT, Spencer JS, Brennan PJ (2004). Continued proteomic analysis *of Mycobacterium leprae *subcellular fractions. Proteomics.

[B15] Marques MA, Neves-Ferreira AG, da Silveira EK, Valente RH, Chapeaurouge A, Perales J, da Silva Bernardes R, Dobos KM, Spencer JS, Brennan PJ, Pessolani MC (2008). Deciphering the proteomic profile of *Mycobacterium leprae *cell envelope. Proteomics.

[B16] Unniraman S, Prakash R, Nagaraja V (2001). Alternate paradigm for intrinsic transcription termination in eubacteria. J Biol Chem.

[B17] Lerat E, Ochman H (2004). Psi-Phi: exploring the outer limits of bacterial pseudogenes. Genome Res.

[B18] Lerat E, Ochman H (2005). Recognizing the pseudogenes in bacterial genomes. Nucleic Acids Res.

[B19] Liu Y, Harrison PM, Kunin V, Gerstein M (2004). Comprehensive analysis of pseudogenes in prokaryotes: widespread gene decay and failure of putative horizontally transferred genes. Genome Biol.

[B20] Stinear TP, Seemann T, Pidot S, Frigui W, Reysset G, Garnier T, Meurice G, Simon D, Bouchier C, Ma L, Tichit M, Porter JL, Ryan J, Johnson PD, Davies JK, Jenkin GA, Small PL, Jones LM, Tekaia F, Laval F, Daffé M, Parkhill J, Cole ST (2007). Reductive evolution and niche adaptation inferred from the genome of *Mycobacterium ulcerans*, the causative agent of Buruli ulcer. Genome Res.

[B21] Madan Babu M (2003). Did the loss of sigma factors initiate pseudogene accumulation in *M. leprae*?. Trends Microbiol.

[B22] Tyagi JS, Saini DK (2004). Did the loss of two-component systems initiate pseudogene accumulation in *Mycobacterium leprae*?. Microbiology.

[B23] Gomez-Valero L, Rocha E PC, Latorre A, Silva F (2007). Reconstructing the ancestor of *Mycobacterium leprae*: The dynamics of gene loss and genome reduction. Genome Res.

[B24] Williams DL, Pittman TL, Deshotel M, Oby-Robinson S, Smith I, Husson R (2007). Molecular basis of the defective heat stress response in *Mycobacterium leprae*. J Bacti.

[B25] Krahenbuhl J, Adams LB, Zwilling BS, Eisenstein TK (1994). The role of the macrophage in resistance to the leprosy bacillus. Macrophage-Pathogen Interactions.

[B26] Hagge DA, Oby Robinson S, Scollard D, McCormick G, Williams DL (2002). A new model for studying the effects of *Mycobacterium leprae *on Schwann cell and neuron interactions. J Infect Dis.

[B27] Takahashi H, Watanabe H (2005). A gonococcal homologue of meningococcal gamma-glutamyl transpeptidase gene is a new type of bacterial pseudogene that is transcriptionally active but phenotypically silent. BMC Microbiol.

[B28] Davids W, Amiri H, Andersson SG (2002). Small RNAs in Rickettsia: are they functional?. Trends Genet.

[B29] Hirotsune S, Yoshida N, Chen A, Garrett L, Suglyama F, Takahashi S, Yagaml K-I, Wynshaw-Boris A, Yoshiki A (2003). An expressed pseudogene regulates the messenger-RNA stability of its homologous coding gene. Nature.

[B30] Ingham C, Hunter IS, Smith MCM (1996). Isolation and sequencing of the rho gene from *Streptomyces lividans ZX7 *and characterization of the RNA-dependent NTPase activity of the over expressed protein. JBC ONLINE.

[B31] Moran NA, Mira A (2001). The process of genome shrinkage in the obligate symbiont *Buchnera aphidicola*. Genome Biol.

[B32] Wilcox JL, Dunbar HE, Wolfinger RD, Moran NA (2003). Consequences of reductive evolution for gene expression in an obligate endosymbiont. Mol Microbiol.

[B33] Hirose T, Sugiura M (2004). Multiple elements required for translation of plastid *atpB *mRNA lacking the Shine-Dalgarno sequence. Nucleic Acids Res.

[B34] Baranov PV, Gurvich OL, Fayet O, Prere MF, Miller WA, Gesteland RF, Atkins JF, Giddings MC (2001). RECODE: a database of frameshifting, bypassing and codon redefinition utilized for gene expression. Nucl Acids Res.

[B35] Truman RW, Krahenbuhl J (2001). Viable *M. leprae *as a research reagent. Int J Lepr Other Mycobact Dis.

[B36] Williams DL, Oby-Robinson S, Pittman TL, Scollard D (2003). Purification of *Mycobacterium leprae *RNA for gene expression analysis from leprosy biopsy specimens. BioTechniques.

[B37] Groathouse NA, Brown SE, Knudson DL, Brennan PJ, Slayden RA (2006). Isothermal amplification and molecular typing of the obligate intracellular pathogen *Mycobacterium leprae *isolated from tissues of unknown origins. J Clin Microbiol.

[B38] Unniraman S, Prakash R, Nagaraja V (2002). Conserved economics of transcription termination in eubacteria. Nucleic Acids Res.

[B39] Rivetti C, Guthold M, Bustamente C (1999). Wrapping of DNA around the *E. coli *RNA polymerase open-promoter complex. EMBO Journal.

[B40] Munteanu MG, Vlahovicek K, Parthasaraty S, Simon I, Pongor S (1998). Rod models of DNA: sequence-dependent anisotropic elastic modeling of local bending phenomena. Trends Biochem Sci.

[B41] Goodsell DS, Dickerson RE (1994). Bending and curvature calculations in B-DNA. Nucleic Acids Res.

[B42] Brukner I, Sánchez R, Suck D, Pongor S (1995). Sequence-dependent bending propensity of DNA as revealed by DNase I: parameters for trinucleotides. EMBO J.

[B43] Hu Y, Coates AR (1999). Transcription of two sigma 70 homologue genes, sigA and sigB, in stationary-phase *Mycobacterium tuberculosis*. J Bacteriol.

[B44] Osada Y, Saito R, Tomita M (1999). Analysis of base-pairing potentials between 16S rRNA and 5'-UTR for translation initiation in various prokaryotes. Bioinformatics.

[B45] Turner DH, Sugimoto N, Jaeger JA, Longfellow CE, Freier SM, Kierzek R (1987). Improved parameters for prediction of RNA structure. Cold Spring Harb Symp Quant Biol.

[B46] Li WH (1993). Unbiased estimation of the rates of synonymous and nonsynonymous substitution. J Mol Evol.

[B47] Pamilo P, Bianchi NO (1993). Evolution of the Zfx and Zfy genes: rates and interdependence between the genes. Mol Biol Evol.

