# Supplementary material for: Implications of high level pseudogene transcription in Mycobacterium leprae
Source: BMC Genomics. 2009 Aug 25;10:397. doi: 10.1186/1471-2164-10-397 (PMC2753549; doi:10.1186/1471-2164-10-397)
Supplement: Additional file 6 — M. leprae pseudogene promoter sequences for in vitro functional promoter analysis. This figure includes the 5'UTRs of M. leprae pseudogenes for cloning into the promoterless reporter vector pGlow-TOPO-TA promoterless reporter-gfp vector for identification of promoter activity. The legend designates which area corresponds to primers for PCR amplification the promoter region including -35 and -10- and initiation site (+1) as well as their position relative to the start codon for the pseudogene. [file 1471-2164-10-397-S6.docx]

**M. leprae |ML2521|ML2521:-Conserved hypothetical protein (pseudogene)**

acggttgtctgcaacattgcttcgtggccgaacttctcaacacaccaggccatgacaaagtggtcgatcacatgcccgatcaccagcaattcgtgaaccaaggtcggcaactctcgttgcgagacaacggtgcgtaccggtttcaagcatgactcaaaactagtcacgcgacgaaccttcggcaacacctgcatttgctacaatgacataataagtgttcacg

**M. leprae |ML1295c|lppL:-Probable conserved lipoprotein LppL (pseudogene)**

gaaatctcctcgacggtaaactcgttgcctgccacatgccggtcaacggtgacagtcatactgtcaagatacctcatcccgaaccggtacatattgtagcatcccagattgaatacaatgatgcaccatcgttccttagaattcgagtttgatgtatagtattttagggttagccgttgctgtcgccggataagcgaacagttcaacactgtttagctggcgtgttgctag

**M. leprae |ML0086c|ML0086c:-Possible phosphotransferase (pseudogene)**

tgtggttgatggcgatgacggccccgccgctggccggcaagttttctataccccgaacactgaccttcagaccctgaatcagccagagcaggcgagcaaactgaattacagtcccgtatagtggttccacagccaattagcttagtgctcctgacggcgcttcgtcgaaacggtcaacaaagagagtaatgctatacattacccgatgcctccaccacagttacctgccgttattagcc

**M. leprae |ML0531|pyrR:-Probable pyrimidine operon regulatory PyrR(pseudogene)**

agggatatttagccagcgatcccgctgggtgccccgcatcgtaaccgggggtcagtagtgctaggctgcccggcagtcgacgtcctttatcgatccgtccggagaggcggagaaggaggtctgcgtaccaccatgtctcgcatgggtgccgcggat gaccccgcttttgccctgggatccagag

**M. leprae |ML0357c|fadE26:-Probable acyl-CoA dehydrogenase FadE26(pseudogene)** tcaccataaggacccagtaagcctgacaaagcggttgctggaattcacactagaacgtgttaatcagcaagacggatggataatcgtttaccgacgtacggtgcccagctttggctagtgatgtggatacgatcgcttaattcaaagaacggctggaatgcgcattagatataaccctgggcaagaggagttgtgtcacgaac

**M. leprae |ML0684|icd1:-Probable isocitrate dehydrogenase Icd1 (pseudogene)**

ccaacgcggttggagggttgccggccagcaagtcggggcTtgacgctggcgctcatgaccgcgttgcagcctatcgcg

tggccacccgacccgacgtcaactctgacagctttagctGgccgtttagcgaccctactagcccatcaaggctgttagtggggcttgcttgacaggcaatagttgacacgggcggagggttttagactctaatgtccaatgaacccaagatcaaagtcgaaggcccggtagtcgaagctcggtggtgat

**M. leprae |ML0211|ML0211:- Possible carboxypeptidase (pseudogene)**

gggtgtaaaggtagcggtctagacgcacccgccccgttttgtggtcgacctcgtacttgttccgctggcctttcggaa

tttcgatggtcacgtcgaattgcaccgtgtcggctccttaaatcttagctaggcccgggtagatcggttacgttgcggagttcaccctagccgacgacgttggtctgaaggctggctcggcacaatggagctagacagtcaggagggctattgaat cctattcgctggcagaaatcttacgtagttgtcggattggcca

**M. leprae |ML1674|ML1674:- 50S ribosomal protein L28 (pseudogene)**

tcatccagcagctgttgaccgtttttggtggttgcccacaacagtcgctatcctgagctggcgcttgggtcagtctaggctgccctggctcccaaatatgtcagttgccggattccagcagaagagttgaggagcattcagaatggccgctgtgtgcgatatctgcgggaaaggtcccggcttcggcaagtcggtatcac

**M. leprae |ML2282c|ML2282c:- Conserved hypothetical protein (pseudogene)**

tattcaaacgcgtaaccagaaaccaaaacggtcgctgacttttggtggcaggtacaggattcgaacctgtgtagcttt

cgcgacggatttacagtccgctcccattggccgctcgggcaacctgccgcctagtagggtacaacgagtgggcagaca

gaacacaaacagttgtcacccgcagaagggacggaccgaatggtggacccatcgttcgccatcgtcagcaagg

**M. leprae |ML0585c|qor:- Probable quinone reductase Qor (pseudogene)**

agaacgagcccggcatcatcgccggcaccagcaacctgctggcgagaacccgccaacacgatgtggggatggaagaggtctggtcgtgcgatcttcatgcccgggcatggataagccacatgcatgcaaccgaaatcgtcgaaactggcgaccctaagtcctgcgctatgtcgaca

**Gray Areas** = Cloning primer (forward & reverse, respectively)

**Yellow Areas** = Predicted promoter region(-35 and -10, respectively)

**Blue Areas = Predicted initiation site (+1)**

**Green Areas** = Predicted pseudogene start Site
